# Blind First-Order Perspective Distortion Correction Using Parallel Convolutional Neural Networks

**DOI:** 10.3390/s20174898

**Published:** 2020-08-30

**Authors:** Neil Patrick Del Gallego, Joel Ilao, Macario Cordel

**Affiliations:** 1 Software Technology, De La Salle University, 2401 Taft Ave, Malate, Manila, Metro Manila 1004, Philippines; 2 Computer Technology, De La Salle University, 2401 Taft Ave, Malate, Manila, Metro Manila 1004, Philippines; joel.ilao@dlsu.edu.ph (J.I.); macario.cordel@dlsu.edu.ph (M.C.II); 3 Data Science Institute, De La Salle University, 2401 Taft Ave, Malate, Manila, Metro Manila 1004, Philippines

**Keywords:** computer vision, distortion correction, image warping, convolutional neural networks

## Abstract

In this work, we present a network architecture with parallel convolutional neural networks (CNN) for removing perspective distortion in images. While other works generate corrected images through the use of generative adversarial networks or encoder-decoder networks, we propose a method wherein three CNNs are trained in parallel, to predict a certain element pair in the 3×3 transformation matrix, $\hat{M}$. The corrected image is produced by transforming the distorted input image using ${\hat{M}}^{-1}$. The networks are trained from our generated distorted image dataset using KITTI images. Experimental results show promise in this approach, as our method is capable of correcting perspective distortions on images and outperforms other state-of-the-art methods. Our method also recovers the intended scale and proportion of the image, which is not observed in other works.

## 1. Introduction

Perspective distortion occurs if the objects in an image significantly differ in terms of scale and position, from how the objects are perceived by an observer [1]. This can be classified as first-order distortions modeled by multiplying an undistorted image with a transformation matrix *M* of size 3×3. First-order distortions can also be caused by an incorrect acquisition environment, such as capturing from an incorrect angle or motions of objects or the photographer. Higher-order distortions are typically caused by capturing a scene with an inappropriate focal length. For example, a wide-angle lens provides a greater angle of view than a normal lens but leads to objects appearing stretched and asymmetrical while the telephoto lens makes objects appear closer to one another than what is perceived in the scene [2].

To some extent, perspective distortion is intentionally applied to images to create artistic effects such as emphasizing a certain object in the scene by making it appear larger than others, and other artistic manipulations and scene editing proposed in the literature [3,4]. Distorted images affect the visual perception of objects in the scene and thus, perspective distortion correction is required on some aspects of photography and computer vision applications.

One area where perspective distortion correction is also needed is in traffic surveillance systems where distorted images affect the performance of vehicle recognition, license plate recognition [5], and other tasks such as speed estimation and distance measurements. Scanned documents may appear warped or misaligned, which need to be corrected for document analysis [6].

**Figure 1:**
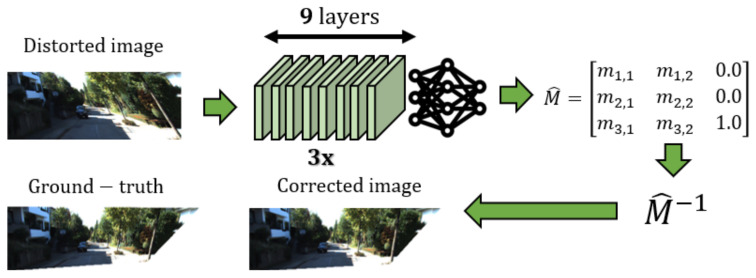
Given a distorted input image, three convolutional neural networks are used for producing $\hat{M}$ transformation matrix that caused the distortion (*M* as ground-truth matrix). The distorted image is transformed to its corrected image by applying ${\hat{M}}^{-1}$.

**Figure 2:**
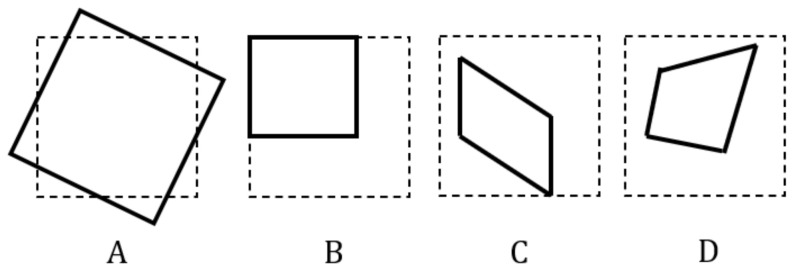
Distortion types that can be corrected by our proposed network. (**A**): rotation. (**B**): Scaling. (**C**): Affine. (**D**): Projective. These are planar transformations identified by Hartley and Zisserman [13]. One or more distortion types may be present in a distorted image.

Image registration algorithms typically use transformation matrices that map an image to a different position or orientation in Euclidean space [7]. In this study, we propose a framework for correcting first-order distortions using multiple convolutional neural networks trained in parallel, that compose the transformation matrix, $\hat{M}$ of size 3×3, of a distorted image, where *M* is the ground-truth that caused the distortion (Figure 1). Distortion types that can be corrected by our proposed network are shown in Figure 2. The key idea to our approach is that we train a certain network to produce a certain element pair in $\hat{M}$, which contributes to a certain effect in the image, i.e., element pair inducing shear effect, or scale effect. ${\hat{M}}^{-1}$ is then applied to the distorted image to produce the correct image. Since each network only produces a certain element pair in $\hat{M}$, it provides a more straightforward approach by simply applying a transformation to correct the image, unlike generating corrected images using GAN or encoder-decoder architectures, which are more difficult to train and prone to instability such as mode collapse [8,9]. While our method requires multiple networks to correct an image, this approach results in a smaller computational footprint because each CNN has a fewer number of hidden layers compared to other architectures [10,11,12] involving deep networks.

We present the following contributions of this study:

- Our network architecture corrects perspective distortion and produces visually better images than other state-of-the-art methods. In terms of pixel-wise reconstruction error, our method outperforms other works.
- Our method, to the best of our knowledge, is the first attempt to estimate the transformation matrix for correcting an image rather than using a reconstruction-based approach. Our method is straightforward and the network design is simpler compared to other works that mainly rely on deep generative models such as GANs or encoder-decoder networks, which are notoriously difficult to train and prone to instability.
- Our method also recovers the original scale and proportion of the image. This is not observed in other works. Recovering the scale and proportion is beneficial for applications that perform distance measurements.

## 2. Related Work

### 2.1. Model-Based Techniques

Some works have been proposed where images are corrected, assuming distortion parameters are provided or available [14,15]. However, there are cases wherein information about the camera lens or acquisition system is unavailable, which inspired some studies on auto-calibration methods where distortion parameters are estimated [16,17,18,19]. Fitzgibbon proposed a single-image automatic distortion correction using a division model to approximate the radial distortion curve [20]. A lightweight auto-rectification method was proposed by Chaudhury et al. [21] where perspective distortions are corrected by performing a RANSAC-based vanishing point detector that restores parallelism of lines in the image. Similarly, the framework proposed by Santana-Cedrés et al. [22] uses a voting scheme for identifying vanishing points and performs perspective correction by simulating camera motion. More recently, an automatic perspective distortion correction for wide-angle portrait images captured on mobile devices was proposed, where a novel face objective term was introduced to properly correct face distortions and background distortions separately [23]. Some works use multiple images with different orientations to properly estimate distortion parameters [24,25,26]. To some extent, methods that combine multiple images for enhancement require some perspective transformation technique [27,28,29,30]. The same technique is implemented for performing image stitching [31,32,33,34,35].

### 2.2. Methods Using Low-Level Features

Using low-level features, such as edges, lines and vanishing points are explored for perspective distortion correction [20,21,22,36,37,38,39]. Wang et al. [18] used an improved Hough Transform for distortion correction while Bukhari and Dailey [19] proposed a sampling method that robustly chooses the circular arcs and determines distortion parameters that are insensitive to outliers. Aside from using low-level features as parameters for distortion correction, assumptions are sometimes included in other studies. For example, images with man-made structures are assumed to appear straight [40]. Lee et al. [41] proposed a set of criteria based on such assumption for upright adjustment of photographs using an optimization-based calibration method. However, methods that rely on low-level features and assumptions do not work well with a variety of images and only work on specialized scenarios. Results from our experiments show that the proposed method of Chaudhury et al. [21] does not correctly rectify our distorted images.

### 2.3. Learning-Based Methods

Blind distortion correction is an ill-posed problem. Therefore, learning-based methods using only a single distorted image are being pursued [10,11,12,42,43,44,45,46]. Deep learning for correcting documents were proposed recently [12,44,45,46] which implements convolutional neural networks, encoder-decoders, and U-net-based architectures [47]. Work on correcting portrait images used an encoder-decoder architecture [10]. The encoder-decoder architecture proposed by Li et al. [11] aims to correct real-world images by predicting the distortion flow and further refining the correction by iterative resampling, which is a predecessor of our work. Instead of using a multi-model network for predicting the distortion flow, we used multiple convolutional neural networks (CNN) that run in parallel to predict the transformation matrix. Our network is trained purely for correcting perspective distortions, unlike the work of Li et al. [11] that correct a wide range of distortion types, such as barrel and pincushion distortions. Furthermore, our results outperforms the method of Li et al. [11], which occasionally generates incorrect rectification of images even on the dataset they have used for training (Places-365 dataset [48]). To some extent, our network properly generalizes to this dataset despite being trained on KITTI [49] images.

## 3. Empirical Analysis on the Transformation Matrix

The motivation behind having networks train in parallel to predict a certain element in *M* is discussed here. An image may be distorted under perspective imaging. A transformation mapping *M* is given by [13]:

(1) $$T\left(\vec{x}\right)=M\vec{x}$$

where *M* is an m×n transformation matrix, where $\vec{x}$ is a vector with *n* entries.

The goal of all the networks is to learn a transformation matrix, given an H×W distorted image $\overset{˘}{I}$. $\overset{˘}{I}$ is generated from H×W original image *I* by creating a random 3×3 transformation *M*, then applying the said transformation for each (x,y) pixel in *I*. Given *M*, $\left(\overset{˘}{x},\overset{˘}{y}\right)$ in $\overset{˘}{I}$ can be represented as:

(2) $$\left[\begin{array}{c} \overset{˘}{x}\\ \overset{˘}{y}\\ \overset{˘}{z} \end{array}\right]=\left[\begin{array}{ccc} m_{1,1} & m_{1,2} & m_{1,3}\\ m_{2,1} & m_{2,2} & m_{2,3}\\ m_{3,1} & m_{3,2} & m_{3,3} \end{array}\right]\left[\begin{array}{c} x\\ y\\ 1 \end{array}\right]$$

Since *M* is homogeneous, $T(\vec{x})$ must be normalized to obtain the inhomogeneous equation [50]:

(3) $$\overset{˘}{x}=\frac{m_{1,1}x+m_{1,2}y+m_{1,3}}{m_{3,1}x+m_{3,2}y+m_{3,3}}\;\;\;\;,\;\;\;\;\overset{˘}{y}=\frac{m_{2,1}x+m_{2,2}y+m_{2,3}}{m_{3,1}x+m_{3,2}y+m_{3,3}}$$

**Figure 3:**
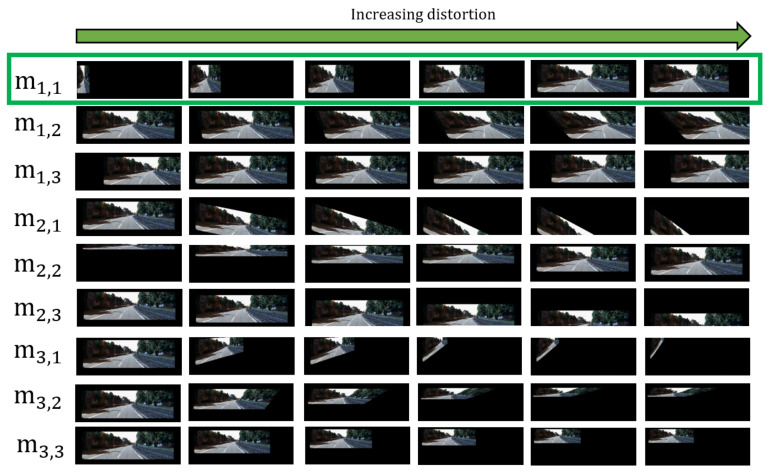
Effects of each element in M to an input image shown frame by frame. The frames for each element in *M* are generated by repeatedly increasing its element values. For example, the frame animations for $m_{1,1}$ are generated by as $m_{1,1}$ increases from 0 to 1 while all other entries in *M* are made constant. The same procedure is performed for creating the animations for the other elements.

Given a single-entry matrix *M* ($m_{3,3}=1$), and an input image *I*, we performed a frame-by-frame analysis on how $m_{i,j}\in M$ (1≤i,j≤3) transforms *I*. In other words, we wanted to visualize the effect of each element in *M* and how these elements contribute to the overall distortion applied to *I*. The frames for $m_{i,j}\in M$ are generated by repeatedly incrementing its element. For example, the frames for $m_{1,1}$ are generated by repeatedly adding Δ to $m_{1,1}$, where Δ is chosen arbitrarily to produce observable frame animations. The origin point for all the frame animations generated is on the top left. Results are visualized in Figure 3.

Based on this experiment, we have identified the element pairs responsible for certain transformation behaviors (e.g., rotating or shearing an image) that a certain network can be trained to estimate. The elements are paired as follows:

- Small changes in $m_{3,1}$ result in a sideways rotation along the Y axis. Small changes in $m_{3,2}$ result in a shearing operation, where the image’s bottom left and bottom right anchor points move sideways and upwards. Equation (3) shows that increasing $m_{3,1}$ and $m_{3,2}$ causes the $\overset{˘}{x}$ and $\overset{˘}{y}$ to shrink. This is represented as an element pair, $\left\{m_{3,1},m_{3,2}\right\}$.
- Based in Equation (3), $m_{1,1}$, $m_{2,2}$, $m_{3,3}$ deal with the scale of the image. The matrix entries, $m_{1,1}$ and $m_{2,2}$, deal with the width and height of the image respectively. Since $m_{3,3}$ is part of the denominator, it changes both the width and height of the image. We do not need to use $m_{3,3}$ as input when training our network because $m_{1,1}$ and $m_{2,2}$ can be inferred instead. This is represented as an element pair, $\left\{m_{1,1},m_{2,2}\right\}$.
- Since $m_{1,2}$ is multiplied by *y* and $m_{2,1}$ is multiplied by *x* in Equation (3), this creates a shearing effect along $\overset{˘}{x}$ and $\overset{˘}{y}$ respectively. This is represented as an element pair, $\left\{m_{1,2},m_{2,1}\right\}$.
- Since no other term is multiplied with $m_{1,3}$ and $m_{2,3}$ in Equation (3), increasing these entries results in pixel-wise displacements along x and y respectively. These are not considered as input for the network as they are typically not observed in distorted images.

Based on this experiment, $m_{1,3}$, $m_{2,3}$ and $m_{3,3}$ can be excluded in training. Thus, Equation (2) can be simplified into the following:

(4) $$\left[\begin{array}{c} \overset{˘}{x}\\ \overset{˘}{y}\\ \overset{˘}{z} \end{array}\right]=\left[\begin{array}{ccc} m_{1,1} & m_{1,2} & 0.0\\ m_{2,1} & m_{2,2} & 0.0\\ m_{3,1} & m_{3,2} & 1.0 \end{array}\right]\left[\begin{array}{c} x\\ y\\ 1 \end{array}\right]$$

The element pairs are used for training the network, which also form the elements in *M* (seen in Equation (4)). Because *M* is invertible, we used *M* as ground-truth and $M^{-1}$ for removing distortion from image $\overset{˘}{I}$.

## 4. Synthetic Distortion Dataset: *d*KITTI

Similar to our predecessor [11] where a synthetic distortion dataset is used, we used the KITTI dataset [49] for populating a set of distorted images and their corresponding *M* that serves as the ground-truth transformation matrix. A distorted image in the dataset has a randomly generated *M* with respect to Equation (4). These images and *M* pairings form the distortion dataset, dKITTI.

**Figure 4:**
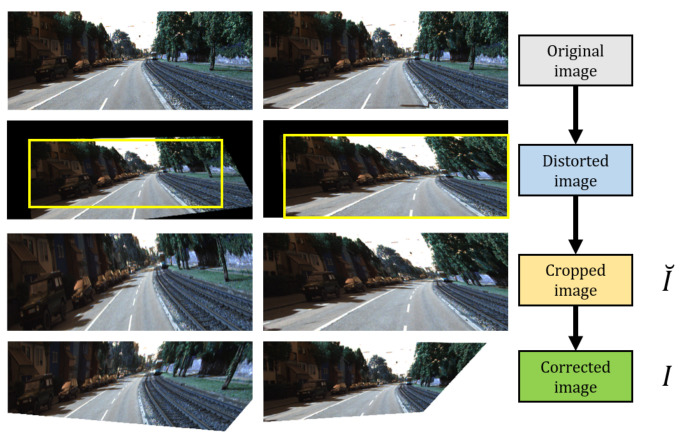
Dataset generation process. The cropped image $\overset{˘}{I}$ is reconstructed based from its estimated transformation matrix inverse. Yellow bounding box shows the region of $\overset{˘}{I}$ in the distorted image. The corrected image, *I*, serves as the ground-truth.

Figure 4 illustrates how we generated dKITTI for training. For each KITTI image, we generated a random *M* for distorting the image and automated the region selection to produce the final distorted image. The range of transformation matrix values (Table 1) used for generating dKITTI images are uniformly sampled. The region selection is performed by fitting a maximum bounding box (Figure 4) which is performed as follows:

1. Declare a bounding box *B* with a size of $\left(B_{w},B_{h}\right)$ in terms of width and height. $\left(B_{w},B_{h}\right)=\left(W_{w},W_{h}\right)$ where *W* refers to the distorted image generated.
2. Iteratively decrease $\left(B_{w},B_{h}\right)$ until the number of zero pixels, *P*, becomes 0. *B* becomes the selected cropped image $\overset{˘}{I}$.
3. Resize $\overset{˘}{I}$ by bilinear interpolation such that $\left({\overset{˘}{I}}_{w},{\overset{˘}{I}}_{h}\right)=\left(O_{w},O_{h}\right)$ where *O* is the original image.

**Figure 5:**
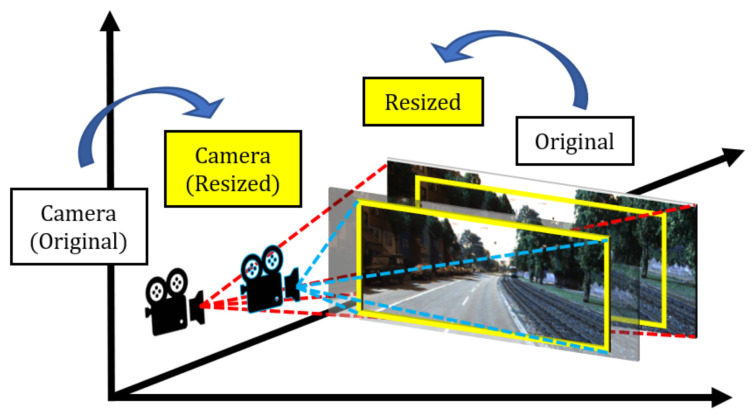
Image projected in 3D space with respect to the camera source. Resizing a region from the original image implies that the camera source moved forward along the *Z* axis.

However, resizing the distorted image, $\overset{˘}{I}$, implies that the 3D positioning of the image has changed and therefore, *M* should be updated. Figure 5 illustrates this observation. $m_{1,1}$ and $m_{2,2}$ deal with the width and height of the image (seen in Figure 3). These elements are updated as follows:

(5) $$m_{1,1}=\frac{B_{w}}{W_{w}}\;\;\;\;,\;\;\;\;m_{2,2}=\frac{B_{h}}{W_{h}}$$

To avoid producing synthetic distorted images that are too extreme or far-fetched from real-world perspective distortions, we further refined our dataset generation by checking if the edge distribution of the distorted and original images are about the same. More specifically, all distorted and original images go through an edge similarity check algorithm (using Sobel operator [51]), where the difference of the total number of edge pixels between the distorted and original images should be less than 25%. This ensures that the loss of overall content from the original image is minimized. Distorted images are regenerated if it does not satisfy this threshold. Figure A3 shows some image samples used for training as well as those that were discarded.

## 5. Proposed Network

Our proposed network consists of three sub-networks which are trained to produce a certain element pair in $\hat{M}$, which forms the transformation matrix that caused the distortion in the input image. The corrected image is obtained by transforming the distorted input image using ${\hat{M}}^{-1}$. More specifically, all three sub-networks require $\overset{˘}{I}$, a cropped distorted image as input (Figure 4), where the goal is to produce $\left\{\hat{m_{3,1}},\hat{m_{3,2}}\right\}$, $\left\{\hat{m_{1,1}},\hat{m_{2,2}}\right\}$ and $\left\{\hat{m_{1,2}},\hat{m_{2,1}}\right\}$ and minimize the difference to $\left\{m_{3,1},m_{3,2}\right\}$, $\left\{m_{1,1},m_{2,2}\right\}$ and $\left\{m_{1,2},m_{2,1}\right\}$ during training. The basis of the element pairs for each network are discussed in Section 3. We refer to these networks as $N(\left\{m_{3,1},m_{3,2}\right\})$, $N(\left\{m_{1,1},m_{2,2}\right\})$ and $N(\left\{m_{1,2},m_{2,1}\right\})$ respectively. This makes training faster and yields better results than having only one network in producing $\hat{M}$. We justify this claim in Section 6.1.

### 5.1. Parallel CNN Model

**Figure 6:**
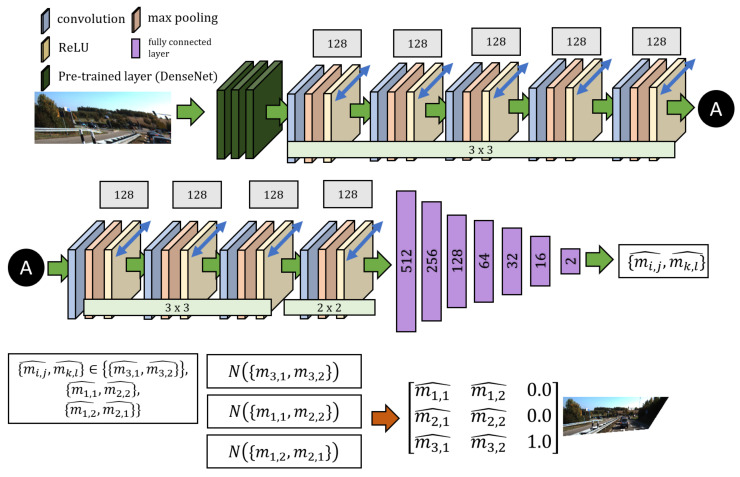
Architectural design of our network. There are three network instances that estimates an element in the transformation matrix $\hat{M}$.

The architectural design of our network is shown in Figure 6. There are three instances of this that attempt to predict element pairs in $\hat{M}$, where each network is trained in parallel. Similarly, the three networks are used in parallel for inference. The CNN accepts an input image of size 1442×575. The input undergoes the pre-trained DenseNet [52] layers, followed by 9 convolutional layers. Each layer uses max-pooling operations and ReLU activations. The last convolutional layer is connected to a fully connected layer which outputs $\left\{\hat{m_{i,j}},\hat{m_{k,l}}\right\}\in \hat{M},i,j,k,l=1,\ldots ,3$.

### 5.2. Training Details

Each network $N(\left\{m_{3,1},m_{3,2}\right\})$, $N(\left\{m_{1,1},m_{2,2}\right\})$ and $N(\left\{m_{1,2},m_{2,1}\right\})$ is trained to minimize the mean square error (MSE) function of its assigned element pair in $\hat{M}$ with respect to element pairs in ground-truth *M*. The total loss function *L* is of the form:

(6) $$L=L_{1}+L_{2}+L_{3}$$

where $L_{1},L_{2},L_{3}$ are defined as follows:

(7) $$L_{1}=\frac{\alpha }{n}\left[\sum_{i=1}^{n}{\left(\left\{m_{1,2},m_{2,1}\right\}-\left\{\hat{m_{1,2}},\hat{m_{2,1}}\right\}\right)}^{2}\right]$$

(8) $$L_{2}=\frac{\beta }{n}\left[\sum_{i=1}^{n}{\left(\left\{m_{1,1},m_{2,2}\right\}-\left\{\hat{m_{1,1}},\hat{m_{2,2}}\right\}\right)}^{2}\right]$$

(9) $$L_{3}=\frac{\gamma }{n}\left[\sum_{i=1}^{n}{\left\{m_{3,1},m_{3,2}\right\}-\left\{\hat{m_{3,1}},\hat{m_{3,2}}\right\})}^{2}\right]$$

where *n* is the number of observed input. The penalty terms α,β,γ, are added to corresponding element pairs based on observed sensitivity conducted from our experiment discussed in Section 3. The following values were used for training: $\alpha =10.0,\beta =1.0,\gamma =1.0\times 10^{6}$. The penalty term, γ, is very large because minuscule differences between $\left\{m_{3,1},m_{3,2}\right\}$ and $\left\{\hat{m_{3,1}},\hat{m_{3,2}}\right\}$ (≥ 1.0 $\times 10^{-6}$ difference) have a noticeable misalignment between ground-truth image *I* and generated image $\hat{I}$.

We implemented the network and performed experiments using PyTorch. The three parallel networks are optimized using ADAM [53] with learning rates set to $5.0\times 10^{-4}$ and batch size of 8.

We trained the networks using an NVIDIA RTX 2080Ti GPU and the networks converge at around 20 epochs. We observed that during training, while some networks converge faster than the others, there were no overfitting incidents. Hence, we let all networks train until all networks have converged to an acceptable loss.

## 6. Evaluation

We evaluated our network architecture using the dKITTI dataset. The network is trained with 95,330 distorted images, while we performed an evaluation on the validation set containing 5018 images. The images are 1442×575 pixels in size.

**Figure 7:**
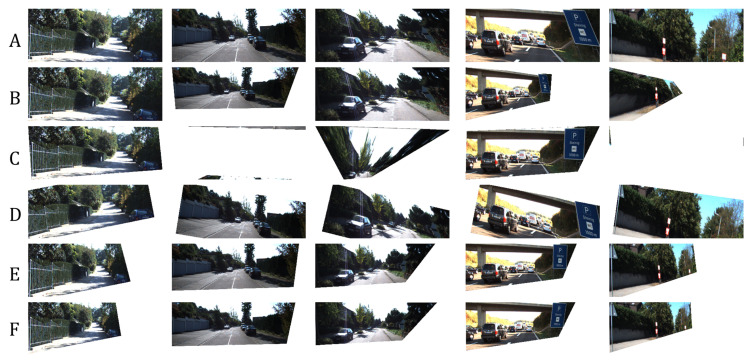
Results using unseen data from KITTI. (**A**): Distorted input images. (**B**): Corrected image using homography estimation (**C**): Corrected image using the technique of Chaudhury et al. [21]. (**D**): Corrected image using the technique of Li et al. [11]. (**E**): Corrected image using our method. (**F**): Ground-truth. Visually comparing the images, our network learned how to correct an image close to the ground-truth compared to other works.

**Figure 8:**
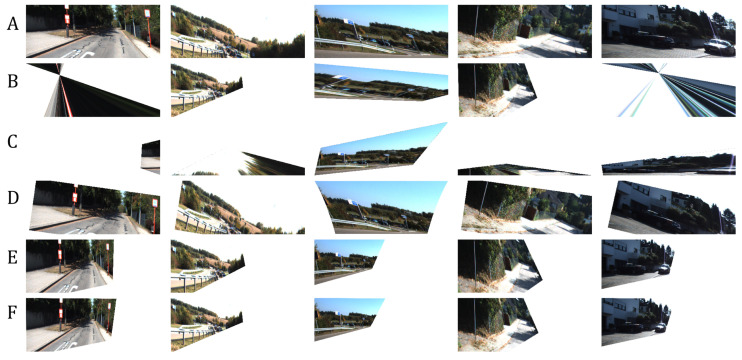
More results using unseen data from KITTI. (**A**): Distorted input images. (**B**): Corrected image using homography estimation (**C**): Corrected image using the technique of Chaudhury et al. [21]. (**D**): Corrected image using the technique of Li et al. [11]. (**E**): Corrected image using our method. (**F**): Ground-truth. Our network can correct an extremely distorted image.

**Figure A1:**
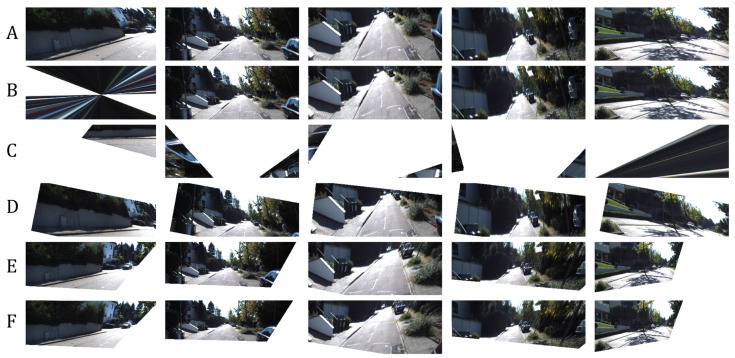
Additional results using unseen data from KITTI. (**A**): Distorted input images. (**B**): Corrected image using homography estimation (**C**): Corrected image using the technique of Chaudhury et al. [21]. (**D**): Corrected image using the technique of Li et al. [11]. (**E**): Corrected image using our method. (**F**): Ground-truth.

**Figure A2:**
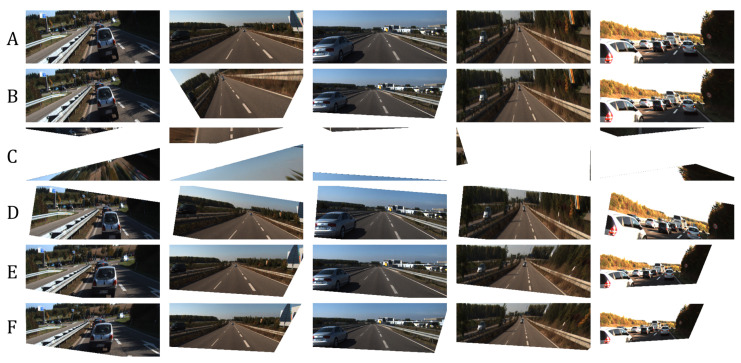
Additional results using unseen data from KITTI. (**A**): Distorted input images. (**B**): Corrected image using homography estimation (**C**): Corrected image using the technique of Chaudhury et al. [21]. (**D**): Corrected image using the technique of Li et al. [11]. (**E**): Corrected image using our method. (**F**): Ground-truth.

**Figure A3:**
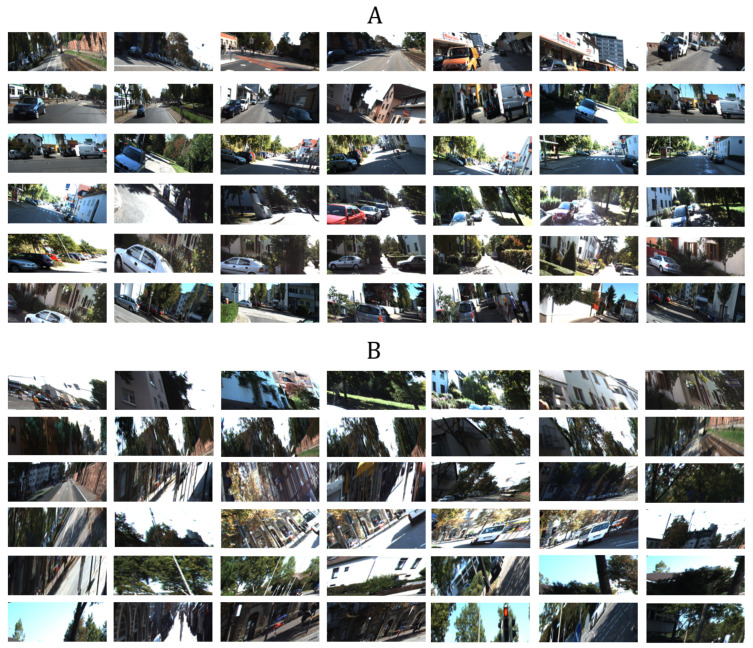
Preview of the dKITTI dataset. (**A**): Some examples of distorted images used for training. (**B**): Some examples of discarded images. Generated distorted images are discarded and regenerated if most of the content from the original image is lost. See Section 4 for details.

We measured the following in terms of transformation matrix error: absolute relative and square relative error and root means squared error (RMSE). The same metrics are used for measuring the pixel-wise error, while structural image similarity (SSIM) [54] is used for checking image reconstruction quality. We also measured the failure rate which is the percentage of images in the validation set that are not properly corrected, such as in the case of homography estimation [55] where it fails to produce visually better images than the input. Performance results are shown in Table 2 and the best results are shown in Figure 7. Figure 8 shows the results of manually picked images that have observable distortion and only depict a small region from the original image. Additional image results are shown in Figure A1 and Figure A2 in the Appendix A.

We compared our method with the following: dataset transformation matrix mean, which is used as a baseline, homography estimation method [55], the methods proposed by Li et al. [11], and Chaudhury et al. [21]. Homography estimation is computed by estimating ${\hat{M}}^{-1}$ for a given distorted image $\overset{˘}{I}$ (Equation (2)) such that the back-projection error to the corrected image *I* is minimized. Homography estimation, however, is not a blind distortion correction technique but this is included for comparison. We used ORB detector [56] for detecting feature points for $\overset{˘}{I}$ and *I* then used RANSAC [57] for minimizing the error. We set a threshold for considering matches only within a certain Euclidean distance, to minimize outliers. For the work of Li et al. [11], we used their pre-trained model, specifically their multi-model distortion network with resampling for generating the corrected image. For the work of Chaudhury et al. [21], we used their independent auto-rectifier algorithm with default parameters.

**Figure 9:**
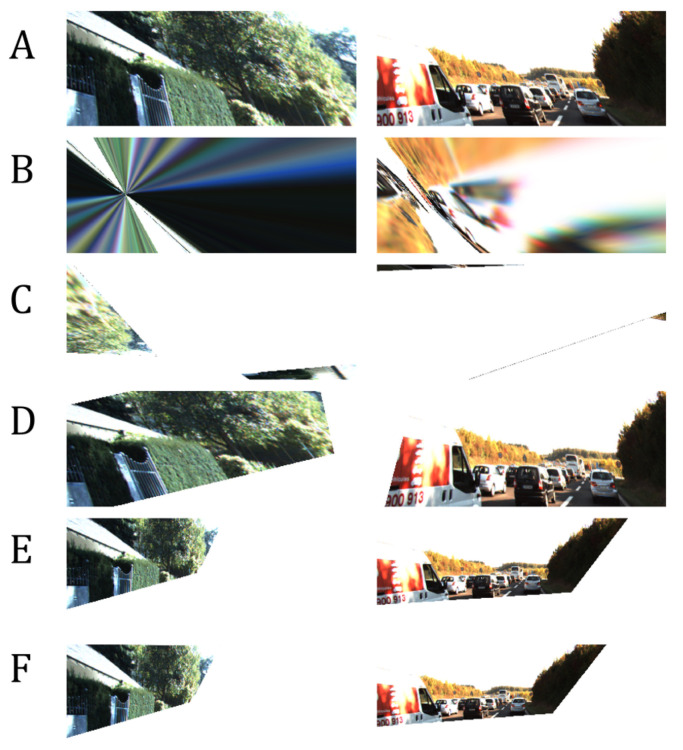
Our network is robust and can still produce a correct image even on extremely distorted images such as when majority of pixels are out of bounds. (**A**): Distorted input images. (**B**): Corrected image using homography estimation (**C**): Corrected image using the technique of Chaudhury et al. [21]. (**D**): Corrected image using the technique of Li et al. [11]. (**E**): Corrected image using our method. (**F**): Ground-truth.

As shown in Table 2, our network architecture outperforms the other methods. To validate the robustness of our network, we input images with extreme distortions, by sampling images with minimum and maximum transformation matrix values in Table 1. Figure 9 show that our network corrects images with extreme distortions and performs better than other methods.

Since the nature of homography estimation involves detecting feature points in the images, there are some occasions wherein there are very few feature points available (incorrect warping observed in Figure 9). Hence, the transformation matrix cannot be inferred properly on some images. In effect, Homography estimation cannot be performed on 13.90% of images in the validation set (specified in Table 2). Our method guarantees that $\hat{M}$ can be inferred on all images in the validation set.

The distortion parameters produced by the methods of Li et al. [11] and Chaudhury et al. [21] have some limitations and can be further improved as follows:

- Both methods do not consider the scaling of images as a possible factor in perspective distortion, unlike our method, as discussed in Section 3.
- Images with low texture and those with shearing, as seen from examples in Figure 8 and Figure 9 affect the correction. This is more observed in the method of Chaudhury et al. [21], which can only handle limited distortions on images. Our method is observed to be robust from these limitations.
- Some images are misclassified as a different distortion type using the method of Li et al. [11]. For example in Figure 8, the third image of row A is misclassified as a barrel or pincushion distortion which resulted in a different corrected image. Our method covers more cases of perspective distortions. As seen in our results, our method consistently produces corrected images.

### 6.1. Experiment on Network Variants

We conducted an experiment to validate the effectiveness of parallel CNNs for perspective distortion correction. The following network variants are described in Table 3. Model A uses DensetNet as the pretrained layer proposed in Figure 6. Model B uses pre-trained ResNet-161 [58] layers instead of DenseNet layers. Model C does not use any pre-trained layer. Model D is similar to Model A except only one instance is trained. The fully connected layer outputs $\left\{\hat{m_{1,1}},\hat{m_{1,2}},\hat{m_{2,1}},\hat{m_{2,2}},\hat{m_{3,1}},\hat{m_{3,2}}\right\}$. The results are summarized in Table 4.

Model C appears to have the lowest transformation matrix error among other variants but the lowest pixel-wise error and highest SSIM accuracy of the corrected images were produced by Model A. The results also show that predicting grouped element pairs and training three network instances in parallel are better than using a single network instance. Hence, Model A is the primary network architecture used for correcting distorted images.

### 6.2. Closeness of Estimations to Ground-Truth

**Figure 10:**
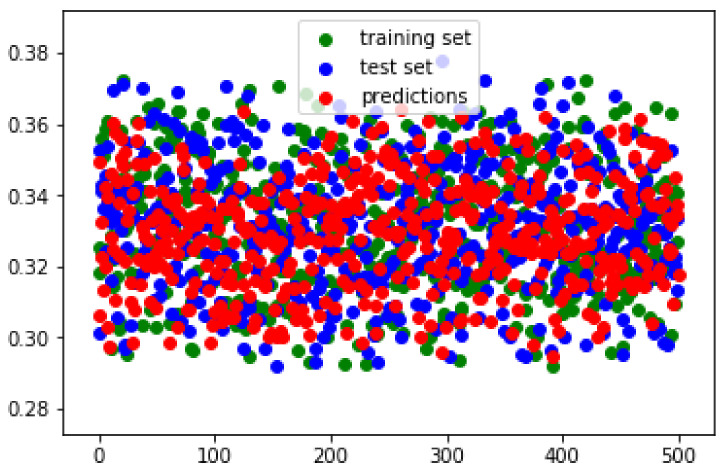
Scatter plot of norm predictions and ground-truth. X axis refers to a certain image number. Y axis is the norm value. The norm of predicted matrices are very close to the training and test set ground-truth matrices.

We randomly selected 500 images each from the training and validation sets, then validated the predicted $\hat{M}$ and compared it against the ground-truth *M*. The norm of $\hat{M}$ and *M* are plotted in Figure 10. Our network predicts shortly by a mean margin of 0.0239 in terms of norm value from the ground-truth. This difference is very small and visually negligible as observed from the image results. The scatter plot also shows that our prediction distribution is almost the same as the ground-truth distribution of the training and test sets.

**Figure 11:**
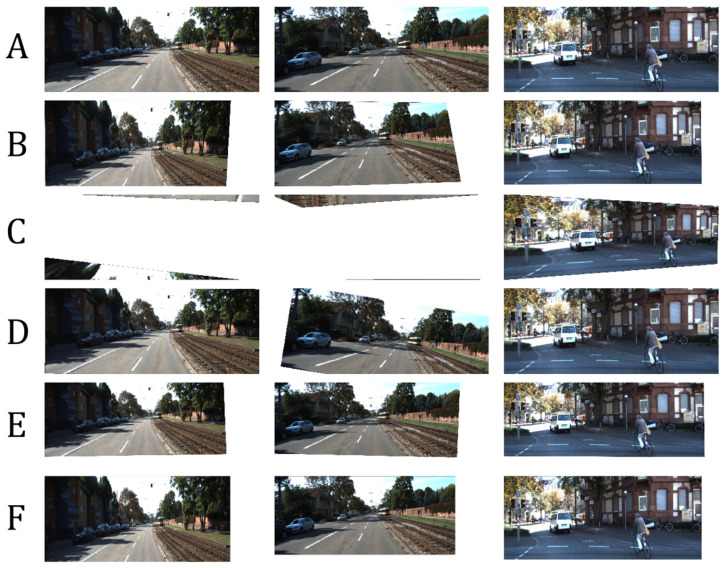
Corrected images with scaling distortion. (**A**): Distorted input images. (**B**): Corrected image using homography estimation (**C**): Corrected image using the technique of Chaudhury et al. [21]. (**D**): Corrected image using the technique of Li et al. [11]. (**E**): Corrected image using our method. (**F**): Ground-truth. Our network can resize the image back to their original scale.

We validated if our network can correct images with different $M_{1,1}$ and $M_{2,2}$ values. As stated in Section 3, these elements deal with scaling of images and should be considered in modelling perspective distortion. We generated 276 distorted images from KITTI where only $M_{1,1}$ and $M_{2,2}$ are uniformly randomized and then used our proposed network for predicting $\hat{M}$. Table 5 summarizes the transformation matrix error and pixel-wise error metrics. The best image results are shown in Figure 11. Based on the results, our network can recover the original scale of the image which cannot be performed by other methods.

### 6.3. Activation Visualization

**Figure 12:**
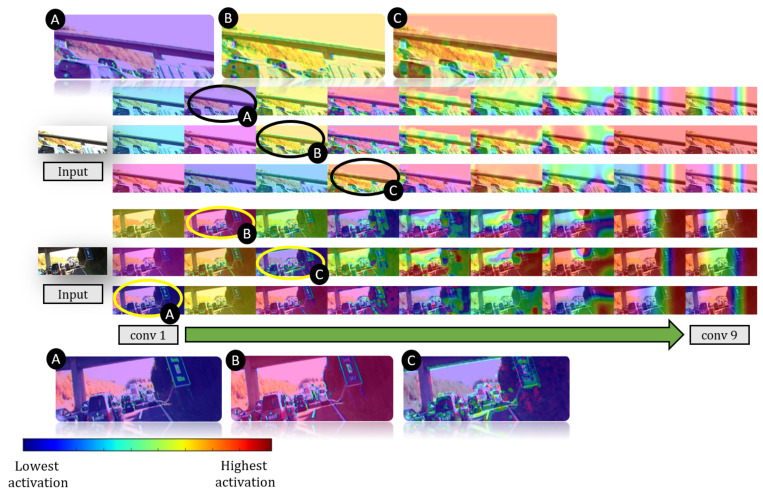
Gradient-weight activation maps for each convolutional layer. Each row represents the networks $N(\left\{m_{3,1},m_{3,2}\right\})$, $N(\left\{m_{1,1},m_{2,2}\right\})$ and $N(\left\{m_{1,2},m_{2,1}\right\})$ respectively. The networks tend to lean towards activation of edges and contours on the first four layers while the remaining layers focus on specific regions. Thumbnails encircled have their zoomed version shown to highlight the activations on earlier layers.

We analyzed how our network behaves by visualizing the gradient-weighted activation maps of the convolutional layers, using the technique of Selvaraju et al. [59]. Figure 12 illustrates the feature maps. As observed in the visualizations, our network tends to extract edges, outlines, then certain regions of the images. The first layer gravitates towards the edges, lines, and contours. For each succeeding layer, the low-level features are being grouped where all edges, lines, and contours appear to be grouped on the 4th layer. Succeeding layers tend to activate on specific regions of the images where the last layer appears to focus on the overall orientation of the image.

### 6.4. Model Generalization

**Figure 13:**
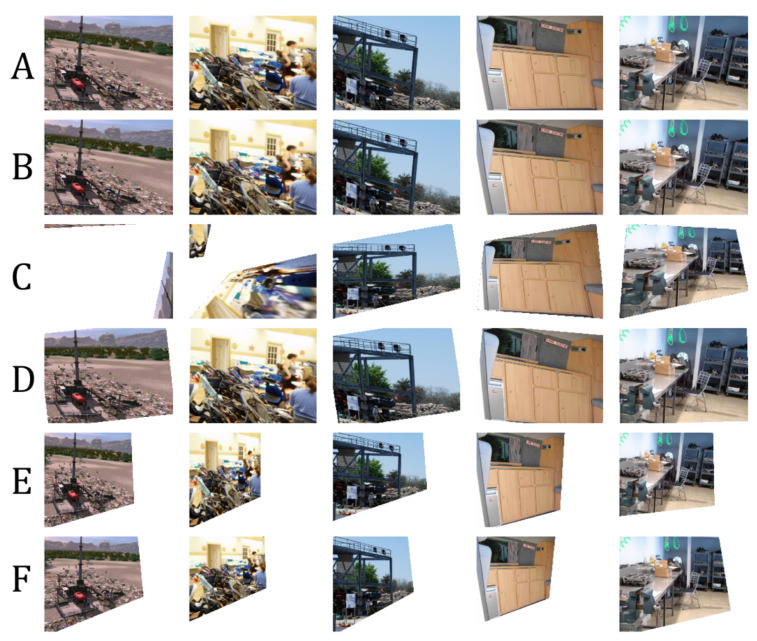
Results using unseen images from Places205 dataset [48]. (**A**): Distorted input images. (**B**): Corrected image using homography estimation (**C**): Corrected image using the technique of Chaudhury et al. [21]. (**D**): Corrected image using the technique of Li et al. [11]. (**E**): Corrected image using our method. (**F**): Ground-truth.

We experimented with our network on unseen data by using test images from Places-205 [48] dataset. A total of 612 images from Places-205 were randomly selected and distorted, where the majority of images have little to no presence of cars and roads. Thus, the images are entirely on a different domain from the KITTI dataset. Table 6 shows the accuracy metrics. Figure 13 illustrates the best results. Notice that our network can still recover the corrected image properly as compared to other methods. While the distortion parameters are similar but the scene context is different, our network can still infer the transformation matrix to correct the image. We speculate that our network is invariant to scene compositions because the activation maps (discussed in Section 6.3) focuses more on edges and lines in the image.

### 6.5. Limitations

**Figure 14:**
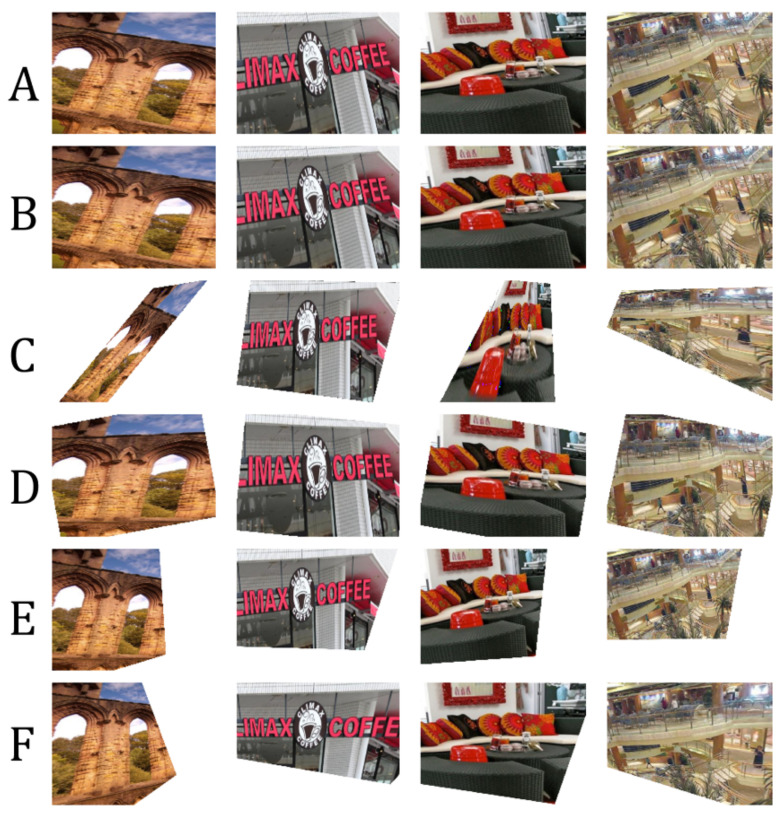
Limitations of our method on unseen images. (**A**): Distorted input images. (**B**): Corrected image using homography estimation (**C**): Corrected image using the technique of Chaudhury et al. [21]. (**D**): Corrected image using the technique of Li et al. [11]. (**E**): Corrected image using our method. (**F**): Ground-truth.

We observed that our network could not properly correct outdoor images with repeating textures as well as indoor scenes with texts or cluttered objects. These examples are shown in Figure 14. Since our network does not recognize specific objects and semantic information in particular, then the network cannot correct images with a dense amount of objects and repetitive textures such as rocks. The network was not trained with any indoor scenes and thus, produces incorrect distortion parameters. We believe that the straightforward solution to this is to retrain the network with more variety of images or perform domain adaptation.

**Figure 15:**
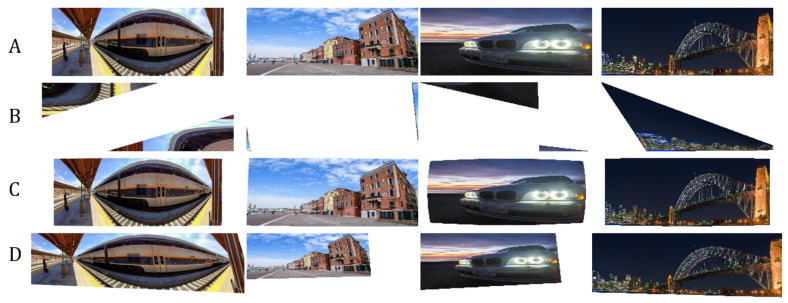
Results of distortion correction using public panoramic images from the Internet. (**A**): Panoramic images. (**B**): Corrected image using the technique of Chaudhury et al. [21]. (**C**): Corrected image using the technique of Li et al. [11]. (**D**): Corrected image using our method. Panoramic images often have a combination of different types of distortions. Our network still attempts to correct the images’ orientation and reduced stretching on some areas. Rightmost image taken by David Iliff (2005).

We also attempted to investigate the limits of our trained network, using panoramic images from the Internet. For an image to be compatible with our network, we either resized the image to 1442×575, assuming the original aspect ratio is preserved, or cropped an area of similar size in the image, with the center as the origin. Results are shown in Figure 15. Panoramic images will most often involve a combination of different distortions, some are higher-order distortions, such as barrel or pincushion distortions. However, results visually show that our network has attempted to correct the images’ orientation and reduced the stretching in some areas as compared to other methods.

## 7. Conclusions

We proposed a blind first-order perspective distortion correction method by using three convolutional neural networks in inferring the transformation matrix for correcting an image where these networks are trained and used in parallel. We discovered that elements in the transformation matrix can be grouped because they perform a specific transformation to the image such as scaling or skewing, which is the rationale behind our approach and design of the network. Our proposed method shows promising results, as shown by outperforming other state-of-the-art methods. Our network can generalize properly on a different domain as well as recover the intended scale and proportion of the image, which could be used for images that appear stretched, making objects in the image appear close to their original scales.

Our network cannot correct images with repeating textures as well as indoor scenes with texts or cluttered objects. We speculate that this could be solved by adding more training samples that cover such cases. We plan to explore how images with higher-order distortions can be corrected, without relying on generative or encoder-decoder architectures which to some extent, was already performed by Li et al. [11] for reconstructing the intermediate flow representation of the distorted image. It would be interesting to use the same strategy (Section 3) that we proposed.

## Figures and Tables

**Table 1:** Range of transformation matrix values used for generating distorted images. The dataset follows a uniform distribution.

	Low	High
$m_{1,1}$ and $m_{2,2}$	$8.0\times 10^{-1}$	$12.0\times 10^{-1}$
$m_{1,2}$ and $m_{2,1}$	$-9.0\times 10^{-3}$	$9.0\times 10^{-3}$
$m_{3,1}$ and $m_{3,2}$	$-7.5\times 10^{-4}$	$7.5\times 10^{-4}$

**Table 2:** Accuracy metrics. Best performance in bold.

Method	Transformation Matrix Error			Pixel-Wise Error/Accuracy				↓ Lower Is Better
	Abs. Rel. ↓	Sq. Rel. ↓	RMSE ↓	Sq.Rel ↓	RMSE ↓	SSIM ↑	Failure Rate ↓	
Dataset mean	$1.92\times 10^{-1}$	$1.52\times 10^{-1}$	$8.60\times 10^{-4}$	0.2665	0.6895	0.5294	0.00%	↑ Higher is better
Homography estimation	2.4457	$1.33\times 10^{1}$	3.1937	0.1838	0.4930	0.6781	13.90%	
Li et al. [11]	N/A	N/A	N/A	$2.32\times 10^{4}$	0.9963	0.0253	0.00%	
Chaudhury et al. [21]	N/A	N/A	N/A	$4.77\times 10^{4}$	0.9975	0.0148	0.00%	
Ours	$7.00\times 10^{-2}$	$3.18\times 10^{-3}$	$5.64\times 10^{-2}$	**0.0361**	**0.2520**	**0.7981**	**0.00%**	

**Table 3:** Network variants experimented.

	Pre-Trained Layer	Instances	Parallel?
Model A	DenseNet	3	Yes
Model B	ResNet-161		
Model C	None		
Model D	DenseNet	1	No

**Table 4:** Accuracy metrics of network variants. Best performance in bold.

Model	Transformation Matrix Error			Model	Pixel-Wise Error/Accuracy			↓ Lower Is Better
	Abs. Rel. ↓	Sq. Rel. ↓	RMSE ↓		Sq.Rel ↓	RMSE ↓	SSIM ↑
Model A	$7.00\times 10^{-2}$	$3.18\times 10^{-3}$	$5.54\times 10^{-2}$	Model A	**0.0361**	**0.2520**	**0.7981**	↑ Higher is better
Model B	$8.15\times 10^{-2}$	$4.34\times 10^{-3}$	$6.59\times 10^{-2}$	Model B	0.0688	0.3580	0.7562	
Model C	$6.89\times 10^{-2}$	$3.13\times 10^{-3}$	$5.25\times 10^{-1}$	Model C	0.0397	0.2707	0.7956	
Model D	$8.19\times 10^{-2}$	$4.56\times 10^{-3}$	$6.75\times 10^{-2}$	Model D	0.0577	0.3270	0.7803	

**Table 5:** Accuracy metrics of the network’s scaling prediction using images with scaling distortion. Our network recovers the scale of images properly. Best performance in bold.

Method	Transformation Matrix Error			Pixel-Wise Error/Accuracy			
	Abs. Rel. ↓	Sq. Rel. ↓	RMSE ↓	Sq.Rel ↓	RMSE ↓	SSIM ↓	
Dataset mean	$1.93\times 10^{-1}$	$2.54\times 10^{-2}$	$1.60\times 10^{-1}$	0.1054	0.5978	0.4886	↓ Lower is better
Homography estimation	1.2790	5.4121	2.3264	0.0968	0.4745	0.4978	
Li et al. [11]	N/A	N/A	N/A	$1.72\times 10^{4}$	0.9969	0.0131	↑ Higher is better
Chaudhury et al. [21]	N/A	N/A	N/A	$5.42\times 10^{4}$	0.9982	0.0051	
Our method	$2.59\times 10^{-1}$	$5.43\times 10^{-2}$	$2.33\times 10^{-1}$	**0.1122**	**0.6339**	**0.6574**	

**Table 6:** Accuracy metrics using Places205 dataset [48]. Best performance in bold. Our network was not trained using images from Places205, but still outperforms other methods.

Method	Transformation Matrix Error			Pixel-Wise Error/Accuracy			
	Abs. Rel. ↓	Sq. Rel. ↓	RMSE ↓	Sq.Rel ↓	RMSE ↓	SSIM ↑
Dataset mean	$2.16\times 10^{-1}$	$2.73\times 10^{-2}$	$1.65\times 10^{-1}$	0.1433	0.5641	0.5805	↓ Lower is better
Homography estimation	2.6514	$1.44\times 10^{1}$	3.7950	0.1522	0.5899	**0.6178**	
Li et al. [11]	N/A	N/A	N/A	$2.19\times 10^{4}$	0.9956	0.0169	↑Higher is better
Chaudhury et al. [21]	N/A	N/A	N/A	$4.35\times 10^{4}$	0.9967	0.0096	
Our method	$3.00\times 10^{-1}$	$6.23\times 10^{-2}$	$2.50\times 10^{-1}$	**0.1355**	**0.5851**	0.6137	

## Appendix A

Additional results are shown in the next figures. The source code for this work is available at: https://github.com/NeilDG/NeuralNets-ImageCorrection. The pre-trained model can be requested by emailing the authors.
